# Editorial

**Published:** 2011-08-16

**Authors:** Usha Mohan Das

**Affiliations:** Editor-in-Chief

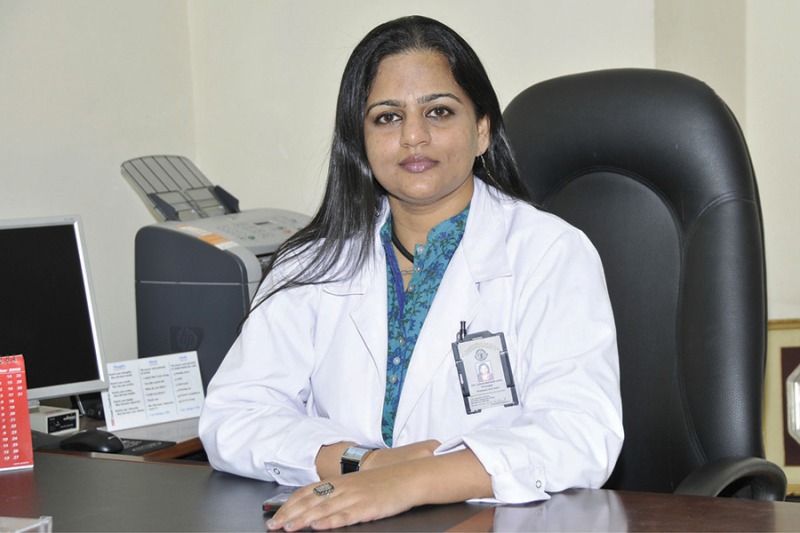



**Of Gizmos and Gadgetry: The Means *vs* the End!**

Technology has been a great value addition in the field of diagnostics and therapeutics in pediatric dentistry. However, the primary challenge still remains managing the child patient ably on the dental chair and eliciting its cooperation.

However, having stated this, I must state that a dental experience today is much more pleasant in view of improved and modified techniques and gadgetry. The ‘Fear’ factor can be minimized or even totally alleviated in many cases because of adjuncts which can be used effectively to minimize chair side time and cut down on procedural intricacies. With the advent of lasers and the inclusion of rotary endodontics, life has become a little less anxious for the practitioner.

Conventional space maintainer therapy needs to be substituted. Do the means always justify the end? A colleague suggested transitional pediatric implants! Even glass fiber reinforced composite resins. Judicious use of general anesthesia and conscious sedation has enabled special care in dentistry. Did I just say ‘judicious’? Its more often than not, a raging debate as to whether convention is to be discarded for the contemporary?

I believe no efforts should be spared toward using gizmos and gadgets at the diagnostic level because the key to good oral health lies in ‘early diagnosis for prevention’. This is one case where I emphasize a personal opinion of sparing no efforts to allow detection or even assumption of a latent disease. It surely solves the numerous problems associated with deforming and debilitating oral/dental afflictions.

Surely, a case wherein I subscribe to the means justifying the end.

**Usha Mohan Das**

Editor-in-Chief

e-mail: ushymohandas@gmail.com

